# Gallic Acid-Loaded Sodium Alginate-Based (Polyvinyl Alcohol-Co-Acrylic Acid) Hydrogel Membranes for Cutaneous Wound Healing: Synthesis and Characterization

**DOI:** 10.3390/molecules27238397

**Published:** 2022-12-01

**Authors:** Abid Naeem, Chengqun Yu, Weifeng Zhu, Xuanbin Chen, Xuan Wu, Lihua Chen, Zhenzhong Zang, Yongmei Guan

**Affiliations:** Key Laboratory of Modern Preparation of Chinese Medicines, Ministry of Education, Jiangxi University of Chinese Medicine, Nanchang 330004, China

**Keywords:** hydrogel membranes, hydrogel patches, wound dressing, polyphenol, wound infection, wound healing

## Abstract

Traditional wound dressings often cannot treat wounds caused by bacterial infections or other wound types that are insensitive to these wound treatments. Therefore, a biodegradable, bioactive hydrogel wound dressing could be an effective alternative option. The purpose of this study was to develop a hydrogel membrane comprised of sodium alginate, polyvinyl alcohol, acrylic acid, and gallic acid for treating skin wounds. The newly developed membranes were analyzed using Fourier transform infrared spectroscopy (FTIR), thermogravimetric analysis (TGA), differential scanning calorimetry (DSC) and scanning electron microscopy (SEM), X-ray diffraction analysis (XRD), sol-gel fraction, porosity, mechanical strength, swelling, drug release and data modelling, polymeric network parameters, biodegradation, and antioxidation (DPPH and ABTS) and antimicrobial activity against Gram-positive and negative bacteria. The results revealed that hydrogel membranes were crosslinked successfully and had excellent thermal stability, high drug loading, greater mechanical strength, and exhibited excellent biodegradation. Additionally, the swelling ability and the porosity of the surface facilitated a controlled release of the encapsulated drug (gallic acid), with 70.34% release observed at pH 1.2, 70.10% at pH 5.5 (normal skin pH), and 86.24% at pH 7.4 (wounds pH) in 48 h. The gallic acid-loaded hydrogel membranes showed a greater area of inhibition against *Pseudomonas aeruginosa*, *Staphylococcus aureus*, and *Escherichia coli* bacteria as well as demonstrated excellent antioxidant properties. Based on Franz cell analyses, the permeation flux of the drug from optimized formulations through mice skin was 92 (pH 5.5) and 110 (pH 7.4) μg/cm^2^·h^−1^. Moreover, hydrogel membranes retained significant amounts of drug in the skin for 24 h, such as 2371 (pH 5.5) and 3300 µg/cm^2^ (pH 7.4). Acute dermal irritation tests in rats showed that hydrogel membranes were nonirritating. Hydrogel membranes containing gallic acid could be an effective option for improving wound healing and could result in faster wound healing.

## 1. Introduction

Human skin functions as the first line of defense of the body to protect itself from threats from the environment, and protects the body from external negative elements [1]. The normal healing process begins whenever the skin is compromised. The process consists of four distinct phases, i.e., homeostasis, inflammation, growth, and finally remodeling to repair the damage. A wound occurs when the dermis’ physiology is disrupted, resulting in damage to the skin’s tissues, irrespective of whether this damage is chronic or acute. Both animals and humans can be treated for burns, injuries, and surgeries as well as slow-healing wounds [2]. The healing of wounds can be a major medical problem, particularly for older populations as well as people suffering from diabetes or obesity. According to reports, the USA invests around 20 billion dollars a year for the treatment and management of chronic wounds [3]. Efforts are being made to develop methods to heal wounds efficiently within a short period of time in order to reduce this financial burden.

A wound infected by bacteria can result in impaired blood flow, prolonged inflammation, and disfigurement after healing [4]. Antibiotics and antiseptics have been around since their discovery to treat wound infections caused by bacteria. Thus, a large proportion of bacteria in the environment have developed resistance to antibiotics, and antiseptics are considered to have potentially harmful effects and limited antimicrobial properties [5]. Thus, it remains necessary to develop new and effective methods for treating wound infections. Therefore, natural antioxidants such as gallic acid can be used to effectively manage burns and wounds through controlled or targeted delivery systems via dermal or transdermal delivery methods [6]. The release of the drug from the dosage form could be adjusted in order to improve its efficacy and frequency of application [7]. The high viscosity of ointments makes them less acceptable to patients. Generally, people report greasy residues and oiling on their garments as the cause of garment soiling. Therefore, it is necessary to develop new formulations for improving patients’ compliance, such as gels, emulgels, and drugs containing microparticles and nanoparticles. Among the most important characteristics of these carriers is their ability to protect the active ingredients and control their release [8].

Currently available wound treatment methods have several disadvantages, including allergic reactions, uncertainty as to the mechanism of action, contamination possibilities, rapid drying of the wound area, and variable results within a batch of products [9]. Presently, conventional dressings such as gauze and bandages are widely utilized in clinics. However, they do not work well for wounds with irregular shapes, deep depths, and narrow widths, or for wounds resulting from arterial ruptures. Furthermore, they are unsuitable for inherently difficult procedures, readily adhere to desiccated wound surfaces, and require surgical and mechanical removal from the site of damage. This can lead to severe consequences. Several pharmaceuticals used to treat wounds have certain limitations, including the potential for causing paranoia, peptic ulcers, hyperglycemia, and slow bone healing. The healing process is also limited by reducing cell mobility to injury sites, inhibiting collagen formation, inhibiting fibroblast proliferation, reducing oxygen flow to the wound site, and reducing immunity. Wound infections can be treated using a variety of methods, such as liquid formulations, topical, systemic, and conventional therapies, as well as traditional and modern wound dressings [10]. Metal–organic frameworks (MOFs) can be used for gas storage, gas separation, sensors, catalysis, and advanced biomedicine (such as wound healing) due to their physicochemical functionality [11,12]. However, MOFs are typically applied directly to wounds as blocks or powders, which limits their practical application [13]. Ideally, wound dressings should not only prevent bacteria from colonizing the wound, but also repair the ECM, prevent bleeding, and maximize surface area and volume for exudate absorption.

Hydrocolloid films, which are moisture-retentive dressings, have become a well-known wound management modality for moist wounds among the large number of modern wound dressings. The hydrogel membrane maintains a moist environment within wound sites, facilitates the removal of wound exudates, has a high-water capacity, and is impermeable to bacteria [14]. Additionally, they prevent excessive amounts of exudate from collecting on the wound bed, as well as facilitate tissue regeneration. In wound dressings, hydrogels are particularly advantageous due to their mild processing conditions and their ability to encapsulate a variety of bioactive agents. In contrast to the traditional topical administration of bioactive molecules, the added molecules can be delivered precisely on time and on site. Hydrogel properties, such as composition, sensitivity to wound stimuli, etc., can vary based on the application. In addition, hydrogels can be used for the delivery of bioactive molecules that assist wound healing, or for the support and maximization of the therapeutic potential of skin or stem cells for angiogenesis and re-epithelialization as well as for the production and maturation of extracellular matrix (ECM) [15]. Many materials have been used in attempts to develop versatile wound dressings, including biopolymers, synthetic polymers, semi-synthetic polymers, and hybrid composite materials [16]. Natural polymers are biocompatible, biodegradable, non-toxic, non-immunogenic, have a high absorption capacity, and have drug delivery properties [17]. Biomaterials can increase patient compliance by extending the therapeutic effect at wound sites through targeted and controlled drug release [18].

Sodium alginate-based interpenetrating (IPN) hydrogels form a porous network with a stable structure. Because of its excellent biocompatibility, the drug is able to be sustained to a maximum degree without compromising its efficacy. Hydrogels based on sodium alginate offer broad potential for sustained and controlled drug release [19]. The hydrophilic nature of PVA allows it to hold water, allowing the wound to stay moist and heal more quickly [20]. In addition to being biocompatible and biodegradable, it also exhibits good mechanical properties. Chemical cross-linking was achieved using ethylene glycol dimethacrylate (EGDMA) in order to develop a physical–chemical hydrogel [21].

The use of bioactive plant compounds in wound healing has gained increasing interest due to their safety and affordability. In particular, gallic acid (GA) is a natural phenolic compound found primarily in fruits, leaves, and wildflowers. GA has been shown to have antioxidant, anti-inflammatory, analgesic, and anti-diabetic properties. GA has been found to improve wound contraction as well as reduce re-epithelialization time in excision wounds. Studies have shown that gallic acid is an effective antibacterial agent against bacteria isolated from wounds. Despite this, gallic acid’s low solubility and susceptibility to environmental factors have limited its application in the pharmaceutical industry. The beneficial characteristics of GA provide the potential for it to be developed into a useful agent for the treatment of wounds [22].

In recent years, hydrogel dressings have received greater attention as wound dressings since they are able to create an ideal environment for the healing of wounds. Hydrogel membranes cover the wound surface, maintain a moist environment, inhibit bacterial growth, allow sufficient oxygen and water to penetrate, are non-adherent and non-allergenic, and can be easily removed without damaging the wound [23]. Therefore, in this study, polymer-based hydrogel membranes consisting of sodium alginate, PVA, acrylic acid, and EGDMA were prepared and characterized by FTIR, TGA, DSC, XRD, SEM, swelling ratio, gel fraction, drug release and kinetic modelling, polymeric network parameters, porosity, mechanical strength, biodegradation, antioxidant activities (DPPH and ABTS), and antimicrobial studies against Gram-positive and Gram-negative bacteria. Furthermore, a dermal irritation study against rat and ex vivo skin permeation study was also performed to determine the drug permeation and retention profile of the hydrogel membrane.

## 2. Results and Discussion

### 2.1. FTIR

**Figure 1:**
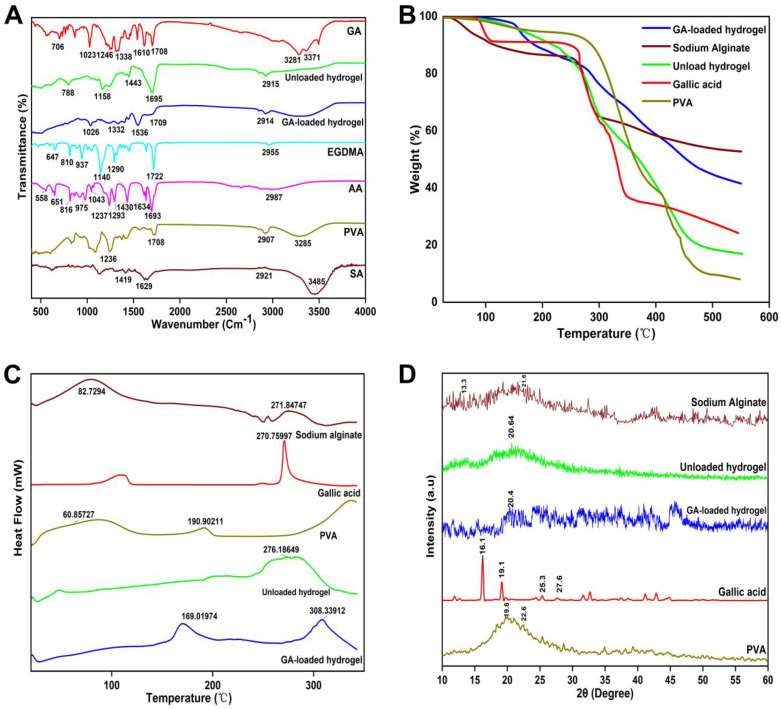
FTIR spectra (**A**), TGA (**B**), DSC (**C**), and XRD (**D**) of prepared hydrogel membranes and their pure components.

FTIR spectra of sodium alginate, PVA, acrylic acid, EGDMA, gallic acid, unloaded and gallic acid-loaded hydrogel membrane are shown in Figure 1A. The FTIR spectrum of sodium alginate showed absorption bands at 3485 cm^−1^ (OH stretching), 1629 cm^−1^ (COO−asymmetric stretching), and 1419 cm^−1^ (COO− symmetric stretching), and is consistent with the results of other researchers [24]. In PVA, OH stretching was observed at 3285 cm^−1^ and C=O vibrations appeared at 1708 cm^−1^ [25]. Asymmetric stretching of C–H group appeared at 2907 cm^−1^ [26]. The absorption band near 3000 cm^−1^ indicates the presence of hydroxyl groups in the acrylic acid, the peak at 1693 cm^−1^ is caused by C=O stretching, and the band at 1634 cm^−1^ represents the C=C stretching [27]. The FTIR spectrum of EGDMA showed a peak at 1713 cm^−1^ corresponding to the stretching vibrations of C=O, and the vibrations at 1633, 1291, and 1153 cm^−1^ are ascribed to the C=C and C–O stretching vibrations of symmetric and asymmetric esters [28]. Blank hydrogel membranes exhibited a different spectrum from their parent components. The vibration bands observed at 1695 cm^−1^ were characterized as peaks of C=O groups, and bands at 2915 cm^−1^ indicated the existence of a -OH group in the structure. The formation of new peaks and functional groups reflects the successful cross linking of the polymers. Furthermore, the FTIR spectrum of a hydrogel membrane loaded with gallic acid was examined to demonstrate the encapsulation of the drug within the membrane. The peaks at 3200 cm^−1^ were located in the same region as those found in the drug spectrum. Additionally, the peaks at 1026 cm^−1^ and 1709 cm^−1^ revealed that gallic acid was physically embedded in the hydrogel membrane.

### 2.2. TGA Study

Hydrogel membrane and other pure compounds were subjected to TGA analysis in order to determine its thermal properties. The thermogram of PVA showed an endothermic event at 180 °C, which is related to the glass transition of the polymer. Another endothermic peak at 250 °C is linked to the decomposition of the samples [29]. The thermal stability of pure sodium alginate was studied by thermogravimetric analysis as shown in Figure 1B. The first weight loss before 100 ℃ was attributed to the loss of absorbed water. With further increasing temperature, the quick weight loss in the temperature range of 200–270 ℃ is associated with the decomposition of sodium alginate [30]. Gallic acid exhibited a two stage degradation that began at temperatures of 230 °C and 320 °C which corresponds to the water loss and degradation of gallic acid. Similarly, in the study of Aydogdu et al., the hydrogel membrane exhibited an initial weight loss of 17% from 25 to 200 °C, and at a temperature of 200–470 °C, the weight loss of 83% was found because of the deformation of bonds in the network and degradation [31].

### 2.3. DSC Study

The PVA thermogram showed a change in the baseline temperature at 60 °C due to glass transition occurring between 49 and 90 °C. It was found that melting occurred between 170 and 200 °C, with a peak temperature of 190 °C. PVA crystallinity was calculated to be 30.7% using the peak integral normalized to the weight of the sample [32]. The DSC thermogram of sodium alginate showed an endothermic peak at 90–100 °C, corresponding to the heat of evaporation of the associated water of hydration, and other peaks representing heat generated during the dissociation of intra- and intermolecular hydrogen bonds within the polymeric matrix, as well as the degradation of the polymer [33]. The sodium molecules had pyrolysis between 220 and 271 °C, and the decomposition rate reached up to the maximum at that point [34]. The DSC thermogram of gallic acid (Figure 1C) showed that its melting endothermic peak was around 270 °C, confirming the crystalline conformation of the material. The thermogram of gallic acid was quite similar to the one in a previous work, which showed a melting endotherm peak of gallic acid at 267 °C [35]. One exothermic peak in the hydrogel membrane was observed at a temperature of 276 °C, indicating that the polymers and monomers were well bonded and that the newly formed structure provided thermal stability to the preparations.

### 2.4. XRD Analysis

The XRD patterns of gallic acid, hydrogel membranes, and other components are shown in Figure 1D. Gallic acid exhibited several notable peaks at diffraction angles of 16.1°, 25.3°, and 27.6°, suggesting crystalline properties [36]. Diffraction patterns of PVA showed peaks at 19.8° and 22.6°, which correspond to crystal plane orientation. According to this diffraction pattern, the PVA chains have strong intermolecular hydrogen bonds, resulting in a crystalline nature [37]. Sodium alginate spectrum indicated two reflections at 2θ = 21.6° and 2θ = 13.3°, which is consistent with the other reported studies. The amorphous nature of the produced unloaded hydrogel membrane can be inferred from the fact that its diffractogram displayed a single, broader, and diffused peak at 2θ = 20.64°. The gallic acid-loaded hydrogel membranes exhibited only one broader peak at 2θ = 20.4°, whereas no other intense peaks of drug were observed in their respective regions. This may be due to the entrapment of the drug within the hydrogel blend, which consequently diminished the crystal lattice properties of the drug.

### 2.5. SEM Analysis

**Figure 2:**
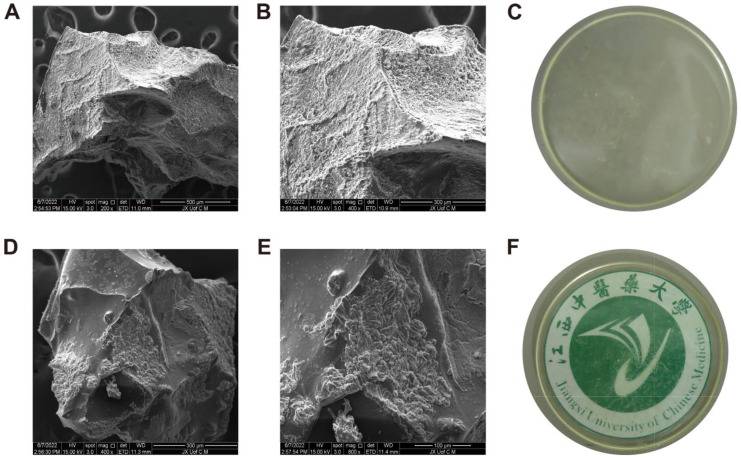
SEM images of hydrogel membranes at magnification of 200× (**A**), and 400× (**B**). Physical appearance of blank hydrogel membrane (**C**). SEM images of hydrogel membranes from different parts of the hydrogel at 400× (**D**), and 800× (**E**). Physical appearance of the hydrogel membrane with the university logo (**F**).

SEM micrographs revealed that the hydrogel membrane consisted of a coarse, wavy, irregular, and dense structure containing both micropores and macropores (Figure 2). Hydrogels with these morphological features show that polymers are successfully cross-linked [38]. Enhanced swelling capabilities are achieved as a result of direct permeation of water within the interstitial spaces and fluid diffusion within the hydrogel network as a result of the porous surface. These pores provide an ideal environment in which encapsulated drugs can be attached and transported locally. Initially, macropores are filled with fluid, followed by micropores gradually absorbing fluid, resulting in increased water absorption by the hydrogel membranes. Fabricated hydrogel membranes exhibit a solid mass and smooth surfaces that confer stability to polymeric networks [39].

### 2.6. Mechanical Properties Analysis

**Figure 3:**
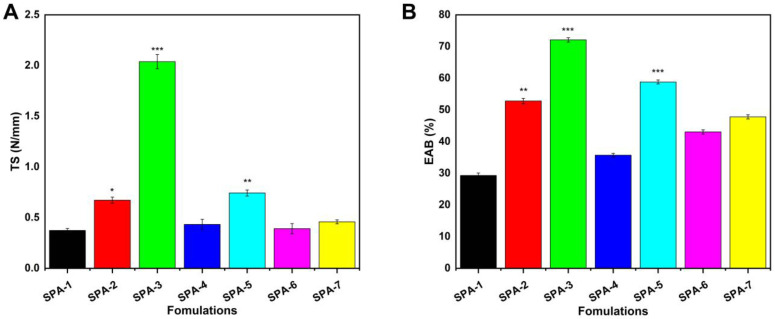
Mechanical properties of hydrogel membranes, such as tensile strength (**A**) and elongation at break (**B**). (Here, * represent *p* value < 0.05, ** *p* < 0.01, and *** *p* < 0.001).

Hydrogel wound dressings must possess certain mechanical properties in order to ensure their durability and strength against external forces. There are several key parameters that must be considered when evaluating hydrogel membranes for wound healing applications, such as their tensile strength and elongation at break. The hydrogels used to cover wounds should be strong and be capable of absorbing frictional stresses without breaking. Increasing the SA content from 1.6 to 3.3% resulted in a higher tensile modulus and increased elongation at break. The increase in cross-linking density is likely to be responsible for this behavior [40]. It was found that the tensile strength of the material also increased with an increase in EGDMA content, as shown in Figure 3.

### 2.7. Sol–Gel Analysis

**Figure 4:**
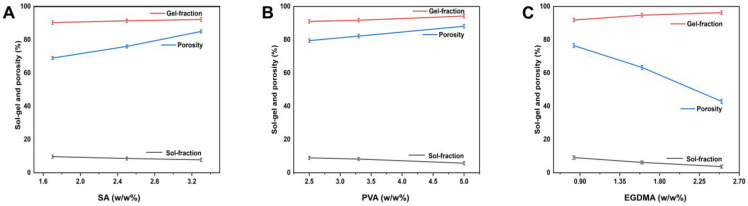
Effect of sodium alginate (**A**), PVA (**B**), and EGDMA (**C**) on sol–gel fraction and porosity of hydrogel membranes.

Gel fraction refers to the crosslinked portion of hydrogels which are formed during the polymerization reaction when the polymers, monomers, and crosslinker reacts together. Sol fraction refers to the non-crosslinked part of the hydrogel. Sol is a relatively small part of the hydrogel formed when high concentrations of at least one component are used, which remains uncrosslinked due to the lack of reactive sites during the polymerization process. Sol–gel analysis was carried out for all hydrogel membranes in order to determine the percentage of crosslinked and uncrosslinked components. Basically, sol–gel analysis measures polymer cross-linking. EGDMA induces gel formation by crosslinking [41]. Therefore, the gel fraction of the hydrogel membrane increases with the increase in EGDMA content. There was a significant increase in the gel fraction as sodium alginate composition increased, but a decrease in the sol fraction, and vice versa. There was a direct relationship between the sol–gel fraction and the concentration of the polymers (Figure 4).

### 2.8. Porosity Study

The porosity of the hydrogel membrane influences swelling ability, drug loading, and release. When pore sizes are larger, swelling occurs more readily, and as a result, more drugs can be loaded and released. Porosity increases as a result of the high viscosity of the reaction mixture which generates interconnected channels. As EGDMA concentration increased (from 0.5 to 1.5 *w*/*w* %), pores and pore sizes were reduced due to the formation of tight junctions and the creation of crosslinked bulk densities that affect the flexibility of the network [42]. The porosity of hydrogel was increased with the sodium alginate concentration was increased, which is consistent with previous studies [43]. The porosity of hydrogel increased with an increase in the amount of PVA, which is also consistent with other studies.

### 2.9. Biodegradation Analysis

**Figure 5:**
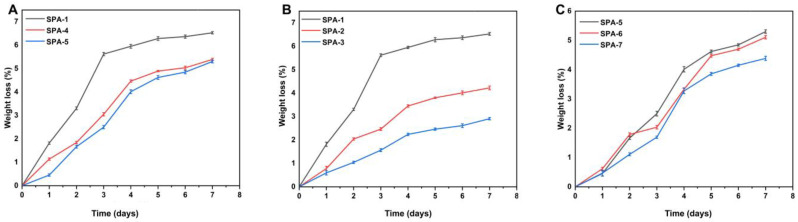
Effect of (**A**) sodium alginate (SPA-1,4,5), (**B**) EGDMA (SPA-1 to 3), and (**C**) PVA (SPA-5 to 7) on biodegradation of hydrogel membranes.

The degradation rate of the prepared hydrogel membranes was assessed at different time intervals as shown in Figure 5. It was found that the degradation speed of hydrogel slowed down with the increase in the amount of EGDMA in the material, which may be attributed to the generation of free radicals within the functional groups. Free radicals play a crucial role in polymerization, since they strengthen the water-gels’ strength, which prevents degradation. Mohamed and his colleagues developed hydrogels using chitosan and PVA that demonstrated a slow degradation rate with increasing concentrations of gel contents [44]. The naturally occurring anionic polysaccharide sodium alginate has been extensively researched and used for a variety of biomedical applications because it is biocompatible, biodegradable, and relatively inexpensive. As the concentration of sodium alginate was increased, the degradation rate of the hydrogel membrane was reduced, which may be attributed to the increase in gel strength.

### 2.10. Swelling Behavior

**Figure 6:**
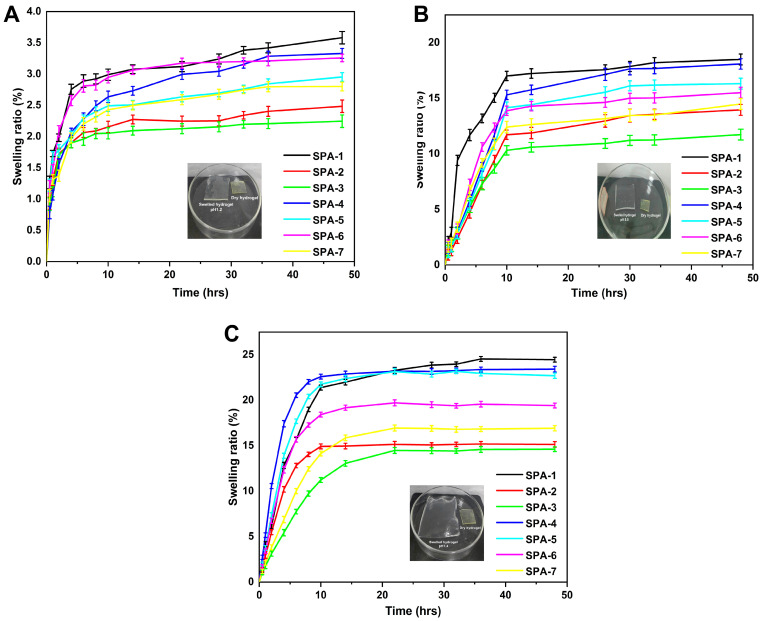
Swelling curve over time of a hydrogel membrane in pH of 1.2 (**A**), pH of 5.5 (**B**), and pH of 7.4 (**C**).

Hydrogel membranes have a large water-holding capacity, which plays a key role in antibacterial functions, wound healing, and other biological applications. Hydrogel membranes are capable of absorbing a substantial amount of wound exudates, making wound healing more efficient and quicker [45]. Several factors influence the swelling behavior of the hydrogel, including hydrophilic groups, crosslink density, polymer network elasticity, as well as pH and temperature within the swelling environment [46]. This implies that the swelling behavior of a hydrogel network is directly influenced by its structure and composition. Hydrogel membranes were prepared with various concentrations of polymers (sodium alginate and PVA), and crosslinker (EGDMA) in order to investigate the swelling ratios in various media and different time intervals. Figure 6 shows the swelling rate of hydrogel membranes at different pH values with time. PVA concentration had a significant impact on the swelling ability of the synthesized hydrogel and the swelling ratio decreased as the PVA concentration was increased. This phenomenon results from the addition of more PVA to the hydrogel membrane in an effort to enhance its mechanical properties [47]. Additionally, sodium alginate–PVA and acrylic acid form an interpenetrating network that enhances the network’s stability. In a similar manner, a decrease in swelling of hydrogels was observed with increasing EGDMA concentrations resulting from increased crosslinking density, which increased the stability of the hydrogel network and reduced the pore size in hydrogels, which limited the penetration of solvents inside the hydrogel [48]. This resulted in a reduced rate of water penetration into hydrogel networks and consequently a reduced rate of swelling [43]. Sodium alginate is pH-sensitive and shows high swelling and release at pH 7.4. The swelling ratio was reduced when the concentration of sodium-alginate was increased a lot, since the gel network became more hydrophilic.

The swelling index of the fabricated hydrogels was as follows: pH 7.4 > pH 5.5 > pH 1.2 [49]. The increased equilibrium swelling ratio (ESR) of fabricated hydrogels at pH 7.4 (ESR of 24.46%) and 5.5 (ESR of 18.49%) compared to pH 1.2 (ESR of 3.58%) in 48 h resulted from the deprotonation of carboxylic functional groups in acrylic acid. This resulted in strong electrostatic repelling forces being generated from the deprotonation of monomers and polymers at pH 7.4 and 5.5. This resulted in the repulsion of the same charged ions; therefore, greater swelling occurred. However, swelling at pH 5.5 and 1.2 was low compared to pH 7.4. This phenomenon is mainly caused by the protonation of functional groups in acrylic acid, sodium alginate, and PVA, which form hydrogen bonds with counterions and are therefore less sensitive to swelling [50]. As a result of strong attractive forces, the charge density of the same group is reduced, and thus hydrogel membranes exhibit a low swelling at pH 1.2 and 5.5 [51]. The results of these studies demonstrated that hydrogel dressings can function as exudate-absorbing wound dressings, resulting in a decrease in infection caused by exudates. The high-water absorption properties of sodium alginate and acrylic acid prevent the collection of liquid on the wound and as a result reduce the risk of infection.

### 2.11. Release and Kinetic Modelling

**Figure 7:**
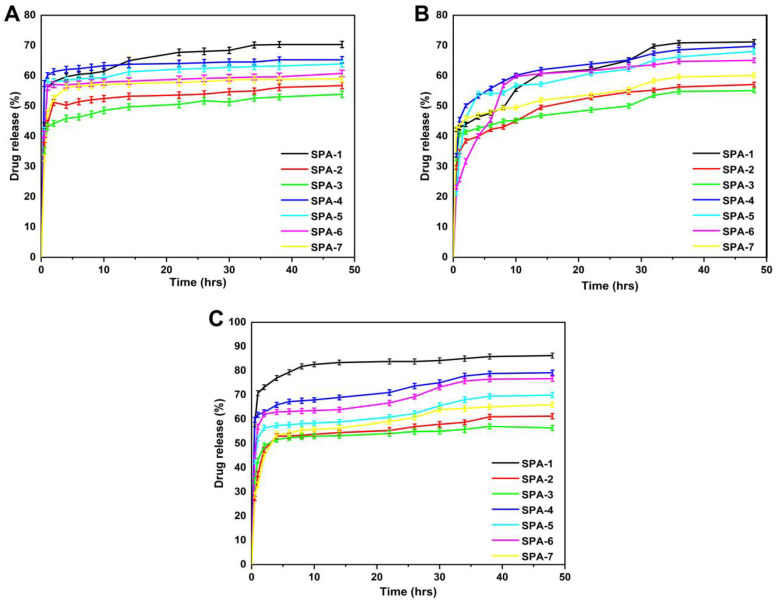
Drug release curve of a hydrogel membrane at pH of 1.2 (**A**), pH of 5.5 (**B**), and pH of 7.4 (**C**).

The amount of gallic acid released (%) from the synthesized hydrogel membrane was tested for 48 h at both an acidic (pH 1.2 and 5.5) and basic pH (pH 7.4) environment taking into account the changes in pH of the skin during wound healing [52]. As shown in the Figure 7A, the percentage of drug release in the buffer at pH = 1.2 ranged from 53.88% to 70.34%. SPA-1 had the highest drug release rate at pH = 1.2 (70.34%), while SPA-3 had the lowest drug release rate at pH = 1.2 (53.88%). Similarly, at normal skin pH value (pH 5.5), the SPA-1 showed a higher release of up to 70.19%, while the lowest release was shown by SPA-2 (57.10%) (Figure 7B). In the buffer with pH = 7.4, the drug release rate was 56.37%~86.24%. SPA-1 had the highest drug release (86.24%), while SPA-3 had the lowest drug release (56.27%). The release curve showed that the release of gallic acid was different in different pH buffers, and the maximum release was in the buffer of pH = 7.4. After 48 h, the maximum release rate of the drug was 86.24% (Figure 7C).

Hydrogel membranes absorb water molecules when immersed in water due to osmotic pressure gradients [53]. Hydrogel membranes swell because of water diffusion, which opens channels, which causes drugs to be released. For the release data, the best fitting model was determined by assessing the regression coefficient value of the model closest to 1 [52]. Table 1 shows the regression coefficients (r) for samples with varying concentrations of sodium alginate (SPA-1, SPA-4, SPA-5), EGDMA (SPA-1, SPA-2, SPA-3), and PVA (SPA-5, SPA-6, SPA-7). These samples followed the Korsmeyer–Peppas release kinetics model because their regression coefficient values were greater than zero order, first order and Higuchi model. The release exponent (n) values of all drug-loaded samples (SPA-1, SPA-2, SPA-3, SPA-4, SPA-5, SPA-6, SPA-7) following the Korsmeyer–Peppas model (release exponent n value of less than 0.5), indicating that the release mechanism was diffusion-controlled.

### 2.12. Structural Parameters of Hydrogel Membranes

Synthesized hydrogel membranes were characterized by their average molecular weight between crosslinks Mc (degree of crosslinking), volume fraction of polymer V2,s (amount of fluid absorbed and maintained by the network), solvent interaction parameter, number of repeating units between crosslinks N, and diffusion coefficient D. The results of the structural parameters are displayed in Table 2. Hydrogels’ maximal absorption and holding capacity can be determined by calculating these characteristics, which indicate the compatibility of the solvent with the polymers used. V2,s and χ values were observed to increase with increasing the EGDMA concentration, indicating the formation of tighter and stiffer gel structures, which is consistent with the results obtained by other researchers [54]. Additionally, Mc and N values decreased as EGDMA concentration increased. This was due to an increase in crosslinking density as a result of an increase in the amount of EGDMA, and vice versa. V2,s indicates swollen polymer and EGDMA concentration indicates degree of swelling.

### 2.13. Antioxidation Analysis

**Figure 8:**
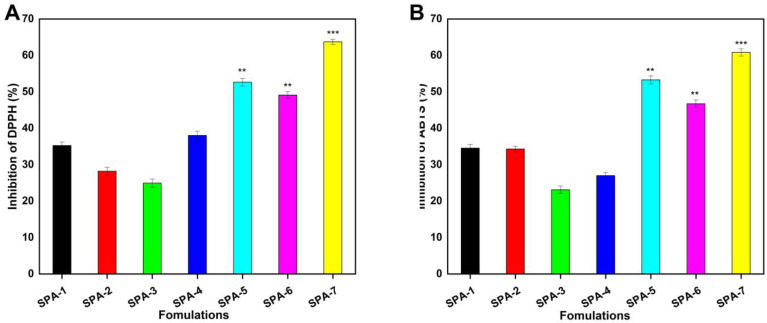
Antioxidant activity of the developed hydrogel membranes against DPPH (**A**), and ABTS (**B**). (Here, ** *p* < 0.01, and *** *p* < 0.001).

The antioxidant activity of the developed hydrogel membranes was evaluated by testing the scavenging efficiency of the hydrogel membranes against DPPH and ABTS (Figure 8). Gallic acid has excellent antioxidant activity, and also has good anti-cancer, anti-mutation, and antibacterial activities. When gallic acid is loaded into the hydrogel membrane, it shows good antioxidant effects [55]. Sodium alginate also possesses antioxidant effects and immune regulatory properties. In the reaction with DPPH, it can eliminate free radicals and thus show antioxidant activity, which is consistent with the experimental results of others [56]. PVA also has an antioxidant activity, for example, food packaging materials synthesized with PVA showed a certain antioxidant activity [57].

### 2.14. Antibacterial Study

**Figure 9:**
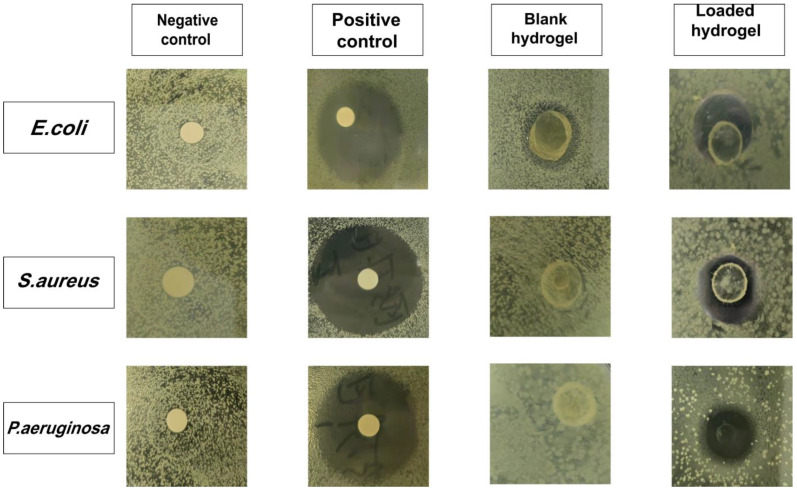
The zones of inhibition for negative control, positive control, unloaded hydrogel and gallic-acid loaded hydrogel membrane against *E. coli*, *S. aureus*, and *P. aeruginosa*.

One of the most challenging issues in wound healing is the risk of bacterial infection, which can lead to serious complications, such as significant pain, fever, and edema. Therefore, the antimicrobial properties of hydrogel membrane are crucial as wound dressings. Therefore, the antibacterial experiment was carried out on Gram-positive and Gram-negative bacteria, and its respective inhibition zones are depicted in Figure 9. No zone development was detected in the negative control and blank hydrogel membrane groups; however, clear zones were found in the positive control (29, 24, and 27 mm) and gallic acid-loaded hydrogel membrane (11, 13, and 14 mm) groups against *S. aureus*, *P. aeruginosa*, and *E. coli*, respectively. Cefepime is an effective antibiotic against both Gram-positive and Gram-negative bacteria [58]. Cefepime was observed to possess antibacterial activity against these bacterial strains, with a smaller zone of inhibition in the case of Gram-negative bacteria as compared to Gram-positive bacteria. This can be observed due to the structure of the bacteria’s cell wall. The cell wall in Gram-negative bacteria is thin and consists of three layers, namely the inside membrane, the peptidoglycan, and the outer membrane. The cell wall of Gram-positive bacteria is thick, but they lack the outermost membrane. This outer membrane acts as a protective layer for Gram-negative bacteria and provides a protective barrier from the external surrounding. Thus, the drug showed better antibacterial activity against Gram-positive bacteria as indicated by the larger zone of inhibition than Gram-negative bacteria. The gallic acid-loaded hydrogel membrane showed effectiveness against both types of bacteria by forming zones of inhibition. The values of anti-bacterial activity were consistent with those reported in the literature for hydrogel membranes containing antibacterial agents. This indicates that the gallic acid in hydrogel was functioning properly and did not encounter any difficulties in leaching out of the membrane. Hydrogels without drug loading also have certain antibacterial effects. For example, sodium alginate, a sustainable biopolymer, has certain antibacterial effects, which is consistent with the results of other people’s studies [59].

### 2.15. Dermal Irritation Study

The skin irritation reactions were assessed at intervals of 1, 12, 24, and 48 h following application of the hydrogel membrane. There were no signs of erythema or edema observed on the skin. There was no skin irritation caused by the synthesized hydrogel membrane, and it appears to be a biocompatible material that is suitable for application as a wound dressing.

### 2.16. In Vitro Permeation and Skin Deposition Study

**Figure 10:**
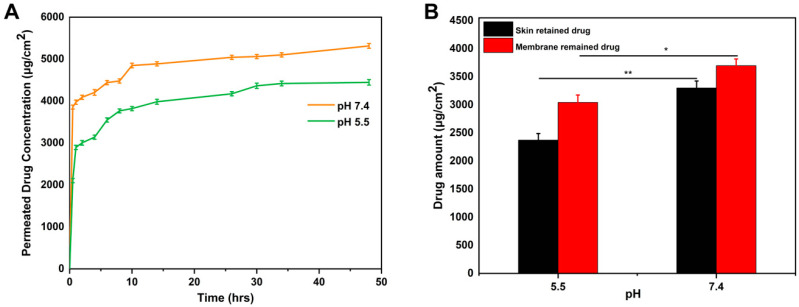
In vitro skin permeation study of optimized hydrogel membrane formulation (SPA-1) at both normal skin pH (5.5) and wounds pH (7.4) (**A**). The amount of drug retained in the hydrogel membrane or deposited on the mice skin is also shown (**B**). (Here, * shows the *p* value < 0.05, and ** *p* < 0.01).

Franz cell apparatus was used to measure both the amount of drug that permeated through the skin and the amount of drug deposited in the skin. Figure 10 shows the amounts of drug permeated through skin from sodium alginate-based (PVA-co-acrylic acid) hydrogel membranes loaded with gallic acid of area 1.5 cm^2^. The developed hydrogel membrane (SPA-1) demonstrated permeation of about 4445 µg/1.5 cm^2^ at pH 5.5 (normal skin pH) and 5314 µg/1.5 cm^2^ at pH 7.4 (wound pH) after 48 h. The permeability coefficient (Kp*10^−3^) calculated was 0.75 and 1.60 cm/h at pH 5.5 and 7.4, respectively. Hydrogels with a loose crosslink structure are reported to permeate more drugs than those with a tight crosslink structure [60].

We conducted kinetic analyses of formulations to determine the pattern of release. In the optimized formulation (SPA-1), the permeability flux was 92 (pH 5.5) and 110 (pH 7.4) μg/cm^2^·h^−1^. Hydrogel membranes release drugs based on a diffusion control mechanism. This formulated hydrogel membrane exhibited controlled release for a period of 48 h. Previous studies using hydrophilic polymers have shown similar results [61]. The amount of gallic acid deposited on the skin of mice and retained in the membrane was 2371 and 3043 µg/cm^2^ (pH 5.5), and 3300 and 3698 µg/cm^2^ (pH 7.4) after 24 h, respectively. Drugs must remain in the skin for an adequate period of time in order to demonstrate their topical pharmacological activity. The results of this study indicate that gallic acid is deposited at a higher concentration than had been reported in earlier studies on hydrogel membranes [62,63]. In addition, polymers with mucoadhesive properties may facilitate biological interactions and internalization. The results indicate that gallic acid has a better residence in the skin.

## 3. Material and Methods

### 3.1. Materials

Sodium alginate from brown algae (SA; M. weight = 216.12 g/moL, Purity ≥ 98) was obtained from Meilune biological company (Dalian, China). Polyvinyl alcohol (PVA; M. weight = 85000–124,000, 87–89% hydrolyzed), acrylic acid (AA; M. weight = 72.06 g/moL), ethylene glycol dimethacrylate (EGDMA; M. weight = 198.22 g/moL), and ammonium persulfate (APS) were purchased from Sigma-Aldrich (Saint Louis, USA). Gallic acid (GA; M. weight = 170.12 g/moL) and sodium bisulfite (SHS) were obtained from Shanghai Aladdin biochemical technology, China. Deionized water was freshly prepared in the laboratory for use throughout the study.

### 3.2. Preparation of Hydrogel Membrane

**Figure 11:**
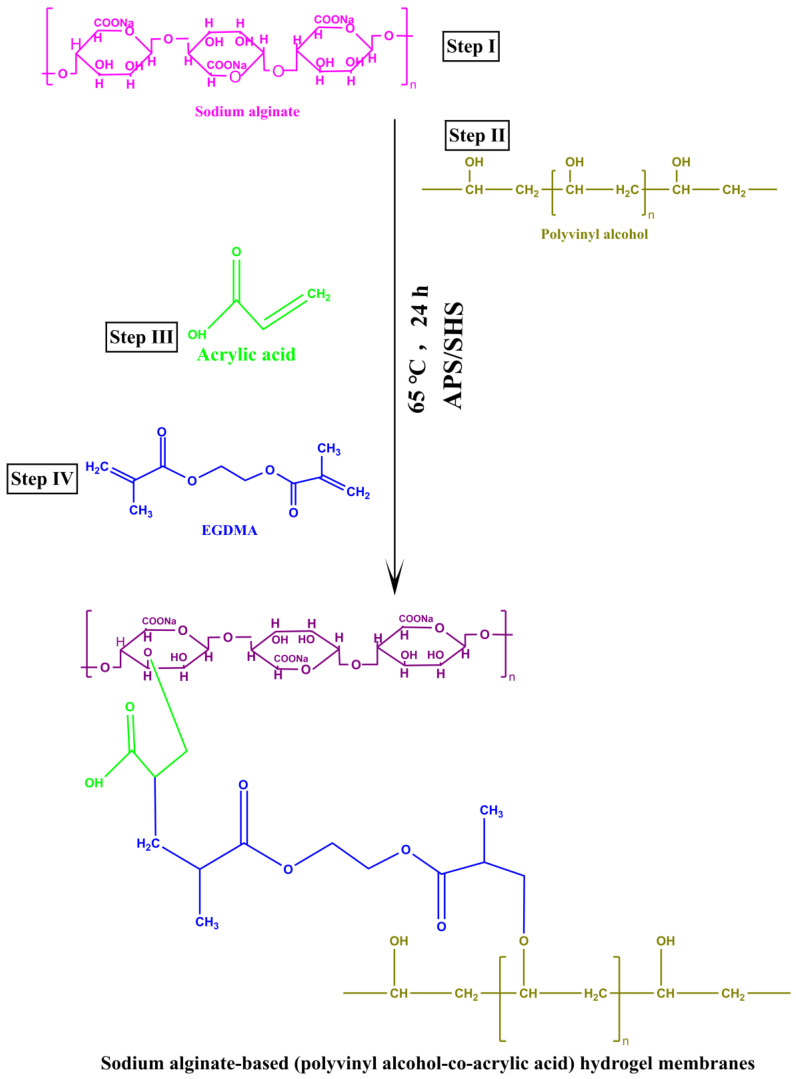
The proposed chemical structure of the synthesized sodium alginate based (PVA-co-acrylic acid) hydrogel membranes.

The free radical polymerization technique was adopted for the preparation of hydrogel membrane with slight modifications [64]. All the formulation components as shown in Table 3, such as PVA, sodium alginate, APS/SHS, acrylic acid, and EGDMA were carefully weighed and a specified amount of water was added. The sodium alginate solution was stirred at 40 °C until it became clear and precipitation-free, while the PVA solution was stirred at 90 °C until it became clear and precipitation-free. After that, the APS/SHS solution was added to acrylic acid and thoroughly blended. This solution was slowly poured into the sodium alginate solution/PVA mixture. After complete mixing, the EGDMA was slowly incorporated into the mixed solution. Finally, a clear solution was obtained after ultrasonication and nitrogen bubbling for 15 to 30 min to remove bubbles. The mixed solution was placed in the petri-dishes in water bath at 40 ℃ for 2 h, 50 ℃ for 4 h, and at 65 ℃ for 18 h. Then, the developed hydrogel membranes were removed from the petri-dishes and washed with a mixture of ethanol:water (70:30) to remove unreacted materials, and finally dried in the oven at 40 ℃ for 2 days. The proposed chemical structure of the developed hydrogel membrane is shown in Figure 11.

#### Drug Loading into the Hydrogel Membranes

Gallic acid was used as a model drug and the process of swelling-diffusion was adopted to load the drug in the hydrogel membrane. First, the gallic acid solution was prepared by dissolving the drug in phosphate buffer of pH 7.4, and then the hydrogel membranes were placed in this drug solution for 48 h. Drug-loaded content was evaluated using the weight difference method i.e., the difference between initial dry weight of hydrogel membrane and final dry weight of hydrogel membrane after drug loading [63].

(1) $$\begin{array}{c} \mathrm{Drug}\;\mathrm{loading}=\mathrm{Drug}\;\mathrm{loaded}\;\mathrm{hydrogel}-\mathrm{Unloaded}\;\mathrm{hydrogel} \end{array}$$

### 3.3. Characterization

#### 3.3.1. FTIR Analysis

FTIR was used to evaluate new bond formation, types, and structures. FTIR analysis of sodium alginate, PVA, acrylic acid, gallic acid, and hydrogel membrane were performed by attenuated total reflectance (ATR) method [65]. The FTIR spectra were recorded at a wavelength of 4000–400 cm^−1^ using a FTIR spectrometer (Spectrum Two, Platinum Elmer).

#### 3.3.2. Thermogravimetric Analysis

Hydrogel membrane and pure materials with a sample of 0.5–5 mg of uniform size were placed in a platinum pan attached to a microbalance. The temperature was increased from 20 to 600 °C at a rate of 20 °C/min (TG/DTA6300, Seiko, Japan) with a 20 mL/min flow of nitrogen [66].

#### 3.3.3. Differential Scanning Calorimetry

Thermal analysis was conducted on both pure materials and hydrogel membranes using a DSC analyzer (Diamond DSC, Perkin Elmer, Waltham, MA, USA) [67]. We heated the samples in a closed aluminum pan at 10 °C/min and purged them continuously with nitrogen gas at 20 mL/min amidst heating. DSC Instruments Universal Analysis 2000 software version 44A was used to analyze all collected data. Data were collected in triplicate (n = 3) and reported as a mean and standard deviation (SD).

#### 3.3.4. X-ray Diffraction Study

X-ray diffraction (TD-3500 X-ray diffractometer, Dandong, China) using Copper Kα examined crystallinity variations over the diffraction angle range of 10 to 60° [68]. Various samples were measured, including sodium alginate, PVA, gallic acid and hydrogel membrane.

#### 3.3.5. Scanning Electron Microscopy

The developed hydrogel membrane was analyzed by scanning electron microscopy (SEM, Quanta 250, FEI company, Eindhoven, The Netherlands). Hydrogel membrane samples were taped to aluminum mounts and then sputtered with gold to form a thin layer. SEM images were captured to determine the surface morphology of hydrogel membranes [69].

#### 3.3.6. Evaluation of Mechanical Properties

Mechanical properties of hydrogel membranes, such as tensile strength (TS) and elongation at break (EAB), were determined at a speed of 1.0 mm/s using a TA.XT plus texture analyzer equipped with a spherical steel probe (P 5S) [70]. The force and displacement generated by the probe during the breaking of membranes were used to calculate the *TS* and *EAB*.

(2) $$\begin{array}{c} TS=\frac{Fm}{Th} \end{array}$$

(3) $$\begin{array}{c} EAB=\frac{\sqrt{D^{2}+R^{2}}}{R}-1 \end{array}$$

where *Fm* is the force that the probe applies to the membrane. *Th* represents the thickness of the membrane. *D* is the amount of displacement which the probe has undergone since its initial interaction with the membrane until the point at which the membrane is broken. *R* represents the radius of the plate.

#### 3.3.7. Sol–Gel Analysis

Sol–gel analysis was used to determine the proportion of soluble, crosslinked, and insoluble components in the hydrogel membrane formulations [62]. In hydrogels, gel represents an insoluble portion, while sol represents the solvent portion. Therefore, sol–gel was studied using the Soxhlet extraction method. Hydrogel membranes of a specified size were cut and placed in a round flask containing deionized water. A condenser was attached to the flask. During the 14-h extraction process, the temperature was maintained at 85 °C. After that, the hydrogel membrane was extracted, placed in an oven to dehydrate completely, and then reweighed. Sol–gel calculations were performed using the equations provided.

(4) $$\begin{array}{c} \mathrm{Sol}\;\mathrm{fraction}\;\%=\frac{M1-M2\;}{M2\;}\times 100 \end{array}$$

where M1 represents the initial weight of the hydrogel before extraction, and M2 indicates the final weight of the dried hydrogel.

(5) $$\begin{array}{c} \mathrm{Gel}\;\mathrm{fraction}=100-\mathrm{Sol}\;\mathrm{fraction} \end{array}$$

#### 3.3.8. Porosity Study

A solvent replacement method was used to assess the porosity of the fabricated hydrogel formulations [71]. The dried hydrogel membranes (Q1) were precisely weighed. They were then immersed in absolute ethanol (purity more than 99.9 percent) for 4 days. After that, the hydrogel membranes were removed, blotted with filter paper to remove the excess solvent, and weighed again (Q2). Similarly, the thickness and dimensions of the membranes were determined. Porosity was determined using the following formula.

(6) $$\begin{array}{c} \mathrm{Porosity}\;\mathrm{percentage}\;\left(\%\right)=\frac{Q2-\;Q1\;}{\rho v\;}\times 100 \end{array}$$

ρ represents ethanol density, whereas V is the volume of the hydrogel following swelling.

#### 3.3.9. Biodegradation Study

The biodegradation study of the hydrogel membrane was conducted in phosphate buffer solution of pH 7.4 at a temperature of 37 ± 0.5 °C [72]. Dried hydrogel membranes were immersed in buffer solutions. They were removed from the buffer solution at intervals of 1, 3, 5, or 7 days and dehydrated at 40 °C in a vacuum oven, weighed again, and returned to the buffer solution. This procedure was performed for a week to assess the rate of biodegradation. The given equation was used to measure the degradation of the hydrogel membrane.

(7) $$\begin{array}{c} D=\frac{K1-K2}{K1} \end{array}$$

where D indicates degradation level, *K*1 shows dry sample weight, and *K*2 is the sample weight following immersion at time (t).

### 3.4. Hydrogel Membrane Network Parameters

The following parameters are important in evaluating the structure and characteristics of hydrogels in swollen condition: the volume fraction of polymer in a swollen form (V2,s), crosslinking junction molecular weights (Mc), solvent interaction (χ), and the number of linkages among crosslinks (N) [73].

The diffusion coefficient (*D*) measures the diffusion rate of a compound along a unit area as a function of the concentration difference over a given time interval. This is determined by the type of polymer and the degree of mobility of its individual segments. According to the following formula, the diffusion coefficient can be estimated.

(8) $$\begin{array}{c} D=\pi {\left(\frac{h.\theta }{4.q_{\mathrm{eq}}}\right)}^{2} \end{array}$$

In this equation, q_eq_ represents the equilibrium swelling of the hydrogel membrane, θ is the slope of the linear part of swelling curves, and h is the thickness before swelling.

Polymer volume fraction, denoted by V2,s, is the proportion of the polymer in its fully swollen condition. Polymer volume fraction was calculated using equilibrium volume swelling (Veq) data of the prepared hydrogel membranes at two different pH values (1.2, and 7.4) using the following equation.

(9) $$\begin{array}{c} V2,s=\frac{1}{\mathrm{Veq}\;} \end{array}$$

Whereas, Mc is useful for estimating the degree to which a polymer network has been crosslinked. It can be calculated using the following formula.

(10) $$\begin{array}{c} \mathrm{Mc}=\frac{\mathrm{dpVs}\left(V^{\frac{1}{3}}2,\;s-V2,\frac{s}{2}\right)}{\ln\left(1-V2,s\right)+V2,s+{\chi V}^{2}2,s} \end{array}$$

where ds and dp refer to the density of the solvent and polymer, respectively. vs represents the solvent’s molar volume and χ represents Flory–Huggins polymer–solvent properties.

The solvent interaction (χ) can be computed using Flory–Huggins theory.

(11) $$\begin{array}{c} \chi =\frac{\ln\left(1-V2,s\right)+V2,s}{V^{2}2,s\;} \end{array}$$

where V2,s represents the equilibrium volume fraction of the swollen gel.

N was determined based on the data of Mc. Calculation of the repeating units between crosslinks was performed using the following equation:

(12) $$\begin{array}{c} N=\frac{2\mathrm{Mc}}{\;\mathrm{Mr}\;} \end{array}$$

Mr refers to the repeating unit’s molar mass. The following equation can be utilized to calculate it.

(13) $$\begin{array}{c} \mathrm{Mr}=\frac{\mathrm{mSAMSA}\;+\;\mathrm{mPVAMPVA}\;+\;\mathrm{mAAMAA}+\mathrm{mEGDMAMEDGMA}}{\mathrm{mSA}+\mathrm{mPVA}+\mathrm{mAA}+\mathrm{mEGDMA}} \end{array}$$

where mSA, mPVA, mAA, and mEGDMA are the masses of sodium alginate, polyvinyl alcohol, acrylic acid, and ethylene glycol dimethacrylate, respectively. MSA, MPVA, MAA, and MEGDMA are the molar masses of sodium alginate, polyvinyl alcohol, acrylic acid, and ethylene glycol dimethacrylate, respectively.

### 3.5. Equilibrium Swelling Ratio (ESR)

ESR was determined at 37 °C using different simulated media, including pH 1.2, pH 5.5, and pH 7.4 [74]. During the experiment, the weight change of hydrogel membranes was evaluated by placing them in their appropriate medium at 37 °C and measuring them at regular intervals. The results were recorded up until the point where there was an equilibrium in terms of the weight of the hydrogel membranes. The following formula was used to determine the percentage increase in the swelling of hydrogel membranes.

(14) $$\begin{array}{c} \mathrm{ESR}=\frac{\mathrm{Xt}-\mathrm{Xi}}{\mathrm{Xi}}\times 100 \end{array}$$

where Xt represents the weight at t × 100 and X_i_ indicates the initial weight of dry hydrogel membranes in grams.

### 3.6. Release and Kinetic Modelling

In vitro drug release from the fabricated hydrogel membranes was evaluated at pH levels of 1.2, 5.5, and 7.4 [75]. Hydrogel membranes containing the drug were immersed in phosphate buffer solutions at pH of 1.2, 5.5, and pH 7.4 inside a USP dissolution type II apparatus at 37 °C and 50 rpm. At regular intervals, aliquots of 5 mL were taken and replaced with a fresh medium of the same volume. Filtered samples were analyzed at a wavelength of 220 nm in triplicate using a UV-Vis spectrophotometer (T6 New Century; Beijing GM).

(15) $$\begin{array}{c} \mathrm{Percent}\;\mathrm{drug}\;\mathrm{release}=\frac{\left(\mathrm{Amount}\;\mathrm{of}\;\mathrm{released}\;\mathrm{drug}\right)}{\left(\mathrm{Amount}\;\mathrm{of}\;\mathrm{loaded}\;\mathrm{drug}\;\right)}\times 100 \end{array}$$

Drug release from hydrogel membranes may be influenced by a variety of factors, including relaxation and swellability of polymer chains, matrix composition, drug nature, and pH of the release medium. As hydrogels exhibit swelling-based controlled release, it is crucial that there is solvent diffusion leading to swelling of the hydrogel and ultimately release of the encapsulated drug. Models of zero order, first order, Higuchi, and Korsmeyer–Peppas were used to determine the pattern of drug release.

(16) $$\begin{array}{c} \mathrm{Zero}-\mathrm{order}\;\mathrm{kinetics}\;\mathrm{Ft}=K0t \end{array}$$

In this case, Ft indicates the amount of drug released at time t, and K0 is the apparent rate constant.

(17) $$\begin{array}{c} \mathrm{First}\;\mathrm{order}\;\mathrm{kinetics}\;\ln\left(1-F\right)=-K1t \end{array}$$

In this formula, F represents the amount of drug that has been released in time t and k1 represents the rate constant.

(18) $$\begin{array}{c} \mathrm{Higuchi}\;\mathrm{model}\;F=K2t^{\frac{1}{2}} \end{array}$$

The amount of drug released at time t is indicated by F, and Higuchi constant K2 is indicated by K2. Several hypotheses form the basis for this model: one, the drug is only diffusing in one direction, and two, the drug’s solubility is lower than the starting concentration in the matrix.

(19) $$\begin{array}{c} \mathrm{Korsmeyer}-\mathrm{Peppas}\;\mathrm{model}\;\frac{\mathrm{Mt}}{M\text{¥}}=K3t^{n} \end{array}$$

where the volume of water absorbed at time t is denoted by Mt, and the amount of water absorbed at equilibrium is indicated by M. The release exponent, n, is a constant that takes into account the gels’ unique geometry and structure. Fickian release mechanism is indicated when n ≤ 0.45, while non-Fickian release mechanism is exhibited when n > 0.45.

### 3.7. Antioxidant Studies

#### 3.7.1. DPPH Antioxidant Activity

The DPPH (2,2-diphenyl-1-picryhydrazyl) free radical scavenging method was used to evaluate the antioxidant activity of the hydrogel membrane [76]. The samples were soaked in methanol for 24 h at an ambient temperature in a darkened room. Then, 2 mL of sample solution was combined with 1 mL of 0.1 mM DPPH methanol solution. Afterward, the mixture was thoroughly mixed and kept in a dark place for 30 min. A UV-Vis spectrophotometer was then used to measure the absorbance of the solution at 517 nm in order to determine the DPPH scavenging activity (DPPH%) according to the following formula.

(20) $$\begin{array}{c} \mathrm{DPPH}\left(\%\right)=\frac{A0-A}{A0}\times 100 \end{array}$$

where A0 and A represent the control and sample absorbance values, respectively.

#### 3.7.2. ABTS Antioxidant Activity

The ABTS assay was used to determine the radical scavenging activity of hydrogel membranes [77]. A solution of 7.4 mM ABTS and 2.4 mM potassium persulfate was mixed at a 1:1 ratio and incubated overnight at room temperature to induce ABTS radicalization. Afterwards, the hydrogel membranes and ABTS solution were mixed together and incubated at 37 °C for 30 min. The absorbance of the solution was measured at a wavelength of 730 nm. The following equation was used to calculate the ABTS scavenging effect.

(21) $$\begin{array}{c} \mathrm{ABTS}\;\mathrm{scavenging}\;\mathrm{effect}\;\left(\%\right)=\frac{A0-A1}{A0}\times 100 \end{array}$$

where A0 represents the absorbance of the ABTS solution, and A1 represents the absorbance of the samples.

### 3.8. Investigation of Antibacterial Activity

Nutrient media was prepared by putting the agar in deionized water and then autoclaving it at 121 °C/15 psi for 30 min. Petri plates were filled with agar media and cooled to room temperature in order to solidify it. After growing the strains for 24 h, swabs of *Staphylococcus aureus* (*S. aureus*), *Escherichia coli* (*E. coli*), and *Pseudomonas aeruginosa* (*P. aeruginosa*) were collected and placed on petri dishes. They were divided into four groups as follows: unloaded-hydrogel membrane, gallic acid-loaded hydrogel membrane, positive control (Cefepime, 1 mg/mL solution), and negative control, which were then applied on the plates accordingly. The zone of inhibition was determined following 24 h of incubation at 37 °C [78].

(22) $$\begin{array}{c} \mathrm{Percentage}\;\mathrm{inhibition}=\frac{\mathrm{Zone}\;\mathrm{of}\;\mathrm{inhibition}\;\mathrm{of}\;\mathrm{test}\;\mathrm{sample}\;\left(\mathrm{mm}\right)}{\mathrm{Zone}\;\mathrm{of}\;\mathrm{inhibition}\;\mathrm{of}\;\mathrm{standard}\;\mathrm{drug}\;\left(\mathrm{mm}\right)}\times 100 \end{array}$$

### 3.9. Acute Dermal Irritation Test

In vivo irritation tests were conducted in accordance with OECD guidelines for testing chemicals for skin irritation, and this experiment was approved by the Institutional Animal Care and Use Committee of Jiangxi University of Chinese Medicine, Nanchang, China (study approval number: JZSYDWLL-20201101). Animal care and operation procedures were in compliance with the China Laboratory Animal Use Regulations, and the animals were handled according to the institutional ethical guidelines. Sprague-Dawley (SD) rats (female, 220 ± 10 g) were used for dermal irritation tests (n = 3 per group) [79]. The dorsal area of the rats was shaved approximately 3 cm × 2 cm with an electric razor. After 24 h, the sodium alginate based (PVA-co-acrylic acid) hydrogel membranes were applied to the backs of the experimental rats, whereas the control animals did not receive any treatment. The skin reactions, including erythema and edema, were evaluated at 1, 12, 24, and 48 h after application.

### 3.10. In Vitro Permeation and Skin Deposition Study

Healthy male Kunming mice (KM), weight of 20 ± 2 g, were provided by Beijing Unilever Co., Ltd. (Beijing, China). The animal experimentation and care procedures were conducted in accordance with the Animal Experimental Center protocol of Jiangxi University of Chinese Medicine, Nanchang, China. KM mice were euthanized, and their skin was excised immediately. Fat and other tissues attached to the skin were carefully removed. Skin samples were kept wet in physiological saline and stored at −20 °C in the freezer prior to experimentation. TP-6 transdermal diffusion tester (Tianjin Jingtuo Instrument Technology Co., Ltd., Tianjin, China) was used for the in vitro skin permeation study [80]. We used Franz cells with a diffusion area of 1.76 cm^2^ and a receptor volume of 15 mL. The rat skin was placed between two compartments with the stratum corneum facing the donor compartment. During the experiment, the cells were clamped, the receptor compartment was filled with a phosphate buffer of pH 7.4, and it was maintained at 37 °C throughout with continuous stirring on a magnetic stirrer at 300 rpm. Hydrogel membranes were placed in the donor compartment over skin. We covered the sampling ports and the donor chambers with parafilm in order to prevent evaporation. Tests were conducted for 48 h. After predetermined time intervals (0.5, 1, 2, 4, 6, 8, 10, 14, 26, 30, 34, and 48 h) a 1 mL sample was removed from the receptor compartment and replaced with 1 mL of fresh buffer solution. The acceptor solutions were analyzed by UV-VIS spectrophotometry at 220 nm. Drug permeation through skin was measured and plotted over time. The flux (J) as well as the permeability coefficient (Kp = flux/concentration in donor compartment) were also determined.

In addition, we determined the amount of drug (gallic acid) retained within the membrane and deposited on the skin after 24 h. Each skin section was cleaned three times with 3 mL PBS and gently dried with filter papers. The skin specimens were cut into small pieces of equal size. The samples were soaked in a pH 7.4 buffer and then subjected to an ultrasonication bath for 3 h, followed by gentle shaking on the orbital incubator platform for 24 h to extract the residual drug. The samples were then analyzed by UV spectrophotometry [63].

### 3.11. Statistical Analysis

All data were presented in the form of mean standard deviation. One-way and Two-way ANOVA and Tukey’s post hoc tests were conducted to determine the degree of statistical variation among the data. Statistical significance between swelling and drug release patterns was determined using a *p*-value calculation, as indicated by * *p* < 0.05, ** *p* < 0.01, and *** *p* < 0.001.

## 4. Conclusions

In this study, sodium alginate-based (polyvinyl alcohol-co-acrylic acid) hydrogel membranes were successfully synthesized using EGDMA as a crosslinking agent by free radical polymerization technique. FTIR, TGA DSC, and XRD methods confirmed hydrogel formation and drug loading (gallic acid), and SEM revealed porous characteristics of the hydrogel membranes. Gel fraction of the hydrogel membranes increased with increasing EGDMA content. The gel fraction also increased with an increase in the concentration of sodium alginate; however, the sol fraction of the hydrogel membrane decreased with the increase in the sodium alginate concentration. Hydrogel membranes demonstrated controlled release for more than 48 h at both acidic (pH 1.2 and 5.5) and basic pH (pH 7.4), showing promise in treating acute and chronic wounds. An in vitro release kinetic study showed that hydrogel membranes followed the Korsmeyer–Peppas model and drug release was diffusion controlled. Drug release times and mechanical properties improved by increasing polymer ratios and monomer concentrations. The gel fraction increased as polymer and crosslinker concentrations were increased. Moreover, the hydrogels showed a high porosity and were biodegradable. DPPH and ABTS tests indicated that the developed hydrogel membranes exhibited good antioxidant activity. Hydrogel membranes were also demonstrated to be effective against both Gram-positive (*E. coli* and *S. aureus*) and Gram-negative (*P. aeuroginosa*) bacteria. These membranes are non-irritating to the skin and improve drug retention in the skin, thereby reducing application frequency. In conclusion, gallic acid-loaded sodium alginate-based (PVA-co-acrylic acid) hydrogel membranes have the potential to play a significant role in the management of skin infections and wounds. This study was mostly limited to in vitro testing. However, more in vitro and in vivo wound healing studies are needed to clarify the mechanism of wound healing effects of the GA-loaded membrane and determine its safety and efficacy profile. Furthermore, the developed hydrogel membranes can be used for the delivery of other flavonoids for the treatment of skin disorders.

## Figures and Tables

**Table 1:** Drug release kinetics of gallic acid-loaded sodium alginate (PVA-co-acrylic acid) hydrogel membranes.

F. Codes	pH	Zero Order		First Order		Higuchi Model		Korsmeyer–Peppas Model	
		K_o_ (h^−1^)	r^2^	K_1_ (h^−1^)	r^2^	K_2_ (h^−1^)	r^2^	r^2^	n
SPA-1	1.2	1.110	0.8624	0.010	0.7153	0.678	0.8577	0.9780	0.314
	5.5	0.5830	0.7336	0.007	0.7548	3.575	0.8801	0.9939	0.133
	7.4	1.250	0.8574	0.047	0.7281	11.598	0.8019	0.9901	0.321
SPA-2	1.2	0.958	0.8555	0.044	0.7136	12.120	0.7775	0.9790	0.346
	5.5	0.422	0.8328	0.005	0.8464	2.486	0.9384	0.9514	0.401
	7.4	0.972	0.9384	0.056	0.7999	11.147	0.8003	0.9702	0.217
SPA-3	1.2	1.456	0.8614	0.065	0.8367	15.360	0.6891	0.9950	0.161
	5.5	0.359	0.7955	0.004	0.8075	2.157	0.9195	0.9481	0.340
	7.4	1.643	0.8831	0.118	0.8455	14.028	0.7269	0.9926	0.082
SPA-4	1.2	0.678	0.8992	0.061	0.7415	14.433	0.8232	0.9780	0.281
	5.5	0.550	0.8370	0.006	0.8553	3.232	0.9393	0.9505	0.409
	7.4	0.755	0.8674	0.096	0.7868	13.002	0.8233	0.9832	0.154
SPA-5	1.2	0.809	0.8875	0.061	0.6595	13.145	0.8126	0.9813	0.476
	5.5	0.501	0.8301	0.006	0.8469	2.952	0.9359	0.9485	0.403
	7.4	0.950	0.8240	0.091	0.7226	12.386	0.8838	0.9793	0.345
SPA-6	1.2	1.004	0.7976	0.047	0.7665	12.331	0.8219	0.9708	0.338
	5.5	0.481	0.7735	0.005	0.7905	2.901	2.9034	0.9905	0.155
	7.4	1.221	0.8954	0.088	0.8004	12.143	0.8627	0.9700	0.275
SPA-7	1.2	1.167	0.7651	0.475	0.8297	17.358	0.9181	0.9919	0.380
	5.5	0.436	0.7936	0.005	0.8080	2.620	0.9177	0.9473	0.337
	7.4	1.726	0.7850	0.656	0.8896	13.529	0.9360	0.9996	0.224

**Table 2:** Flory–Huggins polymeric network parameters of synthesized hydrogel membranes.

F. Codes	V2,s	χ	M_c_	M_r_	N	D × 10^−5^ (cm^2^ s^−1^)
SPA-1	0.051 ± 0.013	0.517 ± 0.035	7322.7 ± 445	77.689 ± 16	188.513 ± 26	0.265 ± 0.025
SPA-2	0.078 ± 0.017	0.527 ± 0.030	3833.3 ± 576	76.372 ± 14	100.384 ± 30	0.252 ± 0.027
SPA-3	0.080 ± 0.020	0.533 ± 0.038	2121.9 ± 654	71.700 ± 15	59.188 ± 19	0.369 ± 0.038
SPA-4	0.094 ± 0.014	0.533 ± 0.032	1605.9 ± 540	80.033 ± 17	39.982 ± 25	0.647 ± 0.061
SPA-5	0.072 ± 0.012	0.525 ± 0.029	7287.7 ± 723	82.300 ± 19	177.100 ± 57	0.251 ± 0.030
SPA-6	0.080 ± 0.010	0.528 ± 0.034	6057.7 ± 589	79.433 ± 20	152.523 ± 43	0.473 ± 0.044
SPA-7	0.109 ± 0.022	0.539 ± 0.041	2258.7 ± 706	78.290 ± 10	57.700 ± 27	0.345 ± 0.032

**Table 3:** Hydrogel membrane feeding composition per 100 g.

Formulations	Sodium Alginate (g)	PVA (g)	Acrylic Acid (g)	APS/SHS (g)	EGDMA (g)	Drug Loaded per 1 g of Hydrogel (g)
SPA-1	1	1.5	26	0.4/0.4	0.5	0.617
SPA-2	1	1.5	26	0.4/0.4	1	0.332
SPA-3	1	1.5	26	0.4/0.4	1.5	0.317
SPA-4	1.5	1.5	26	0.4/0.4	0.5	0.514
SPA-5	2	1.5	26	0.4/0.4	0.5	0.501
SPA-6	1.5	2	26	0.4/0.4	0.5	0.488
SPA-7	1.5	3	26	0.4/0.4	0.5	0.414

## Data Availability

The data is contained within the article.

## Sample Availability

Samples of the compounds are available from the authors upon reasonable request.
